# Pin1 Promotes Regulated Necrosis Induced by Glutamate in Rat Retinal Neurons via CAST/Calpain2 Pathway

**DOI:** 10.3389/fncel.2017.00425

**Published:** 2018-01-22

**Authors:** Shuchao Wang, Lvshuang Liao, Mi Wang, Hongkang Zhou, Yanxia Huang, Zhen Wang, Dan Chen, Dan Ji, Xiaobo Xia, Yong Wang, Fengxia Liu, Jufang Huang, Kun Xiong

**Affiliations:** ^1^Department of Anatomy and Neurobiology, School of Basic Medical Sciences, Central South University, Changsha, China; ^2^Department of Ophthalmology, Xiangya Hospital, Central South University, Changsha, China; ^3^Department of Forensic Science, School of Basic Medical Sciences, Central South University, Changsha, China; ^4^Department of Human Anatomy, School of Basic Medical Science, Xinjiang Medical University, Ürümqi, China

**Keywords:** glutamate, regulated necrosis, calpain2, calpastatin, Pin1

## Abstract

The purpose of the current study was to investigate whether peptidyl-prolyl *cis/trans* isomerase NIMA-interacting 1 (Pin1) can interact with calpastatin (CAST) and regulate CAST/calpain2, under excessive glutamate conditions, and subsequently regulate necrosis in rat retinal neurons. Glutamate triggered CAST/calpain2-mediated necrosis regulation in primary cultured retinal neurons, as demonstrated by propidium iodide-staining and lactate dehydrogenase assay. Co-IP results and a computer simulation suggested that Pin1 could bind to CAST. Western blot, real-time quantitative polymerase chain reaction, immunofluorescence, and phosphorylation analysis results demonstrated that CAST was regulated by Pin1, as proven by the application of juglone (i.e., a Pin1 specific inhibitor). The retinal ganglion cell 5 cell line, combined with siRNA approach and flow cytometry, was then used to verify the regulatory pathway of Pin1 in CAST/calpain2-modulated neuronal necrosis that was induced by glutamate. Finally, *in vivo* studies further confirmed the role of Pin1 in CAST/calpain2-modulated necrosis following glutamate excitation, in the rat retinal ganglion cell and inner nuclear layers. In addition, a flash electroretinogram study provided evidence for the recovery of impaired visual function, which was induced by glutamate, with juglone treatment. Our work aims to investigate the involvement of the Pin1-CAST/calpain2 pathway in glutamate-mediated excitotoxicity.

## Introduction

Glutamate, which is one of the twenty essential amino acids, is the main physiological excitatory neurotransmitters in the central nervous system (CNS) (Veyrat-Durebex et al., 2016; Yan et al., 2016). After release from the presynaptic terminal, glutamate is quickly cleared by diffusion and uptake, mediated by glutamate transporters (Bergles and Jahr, 1998; Grewer et al., 2008; Afzalov et al., 2013). Glutamate transporters control signal transmission at glutamatergic synapses, initially by buffering, and subsequently by removing glutamate from the synaptic cleft into adjacent neurons and glia cells (Afzalov et al., 2013; Madji Hounoum et al., 2017). In addition to uptake, these transporters are involved in release of glutamate in a process termed “reversed uptake” (Rossi et al., 2000; Grewer et al., 2008). All of these processes maintain the glutamate concentration in the synaptic cleft, regulate activation of high-affinity extrasynaptic glutamate receptors (Diamond and Jahr, 1997; Fang et al., 2010). It should be noting that, glial cells play critical roles in neurons survival and function in numerous ways, such as maintenance of synaptic glutamate homeostasis and modulation of neuronal susceptibility to glutamate excitotoxicity, or lactate-mediated metabolic coupling (Veyrat-Durebex et al., 2016; Madji Hounoum et al., 2017).

Excitotoxicity was a toxic effect of excessive or prolonged activation of receptors by excitatory amino acids, first proposed by Dr. Olney (Olney and Sharpe, 1969; Wang and Qin, 2010). Failure to remove glutamate after synaptic release and activation of reversed uptake (e.g., under some injury conditions) can lead to glutamate accumulation in the extracellular space, leading to uncontrolled continuous depolarization of neurons, an excitotoxicity process (Afzalov et al., 2013; Kritis et al., 2015). Glutamate receptors are classified into three ionotropic classes [*N*-methyl-D-aspartic acid receptor (NMDA), α-amino-3-hydroxy-5-methyl-4-isoxazole propionate receptor (AMPA), and kainic acid receptors] and three metabotropic classes (Wang and Qin, 2010; Madji Hounoum et al., 2016). Ionotropic receptors are ligand-gated ion channels that open upon the binding of glutamate, leading to the influx of calcium (Wang and Qin, 2010; Madji Hounoum et al., 2016). Activation of metabotropic receptors, which are the G-protein coupled receptors, leads to release of calcium from intracellular stores (Wang and Qin, 2010; Madji Hounoum et al., 2016). AMPA and NMDA receptors are largely responsible for calcium flux across neuronal cell membranes (Hollmann et al., 1991; Madji Hounoum et al., 2016). Under pathological stimuli, excessive glutamate over-activates glutamate receptors, resulting in an increased intracellular calcium influx (Kritis et al., 2015). Increased intracellular calcium disrupts calcium homeostasis and triggers a cascade of signaling pathways, such as proteases activation, energy deficiency, and oxidative stress, which leads to excitotoxic neuronal death, i.e., necrosis, apoptosis, and regulated necrosis (Xu et al., 2007; Leon et al., 2009; Del Rosario et al., 2015). Regulated necrosis is a form of programmed cell death that is mainly divided into necroptosis, ferroptosis, parthanatos, etc., which exhibits morphological features that are similar to those of necrosis, including plasma integrity loss and organelle swelling (Galluzzi et al., 2014; Pasparakis and Vandenabeele, 2015; Liao et al., 2017; Kong et al., 2017; Wang Z. et al., 2017). During the last years, the development of drugs targeting pathophysiological mechanisms of glutamate-induced excitotoxicity is available to slow the progression of the disease (Blasco et al., 2016; Madji Hounoum et al., 2016). However, currently, the molecular pathway underlying excessive glutamate modulated neuronal regulated necrosis is not clearly understood (Fulda, 2013; Zhang et al., 2013).

Primary cultures of retinal neurons and cultures of retinal neuron lines are used to study the molecular pathways of neurotoxicity induced by glutamate (Petegnief et al., 2001; Maher and Hanneken, 2005; Oliveira et al., 2011; Miao et al., 2012; Santos-Carvalho et al., 2013; Morgan-Warren et al., 2016). Because both ionotropic and metabotropic glutamate receptors are exist in primary retinal neurons and retinal neuron lines (Connaughton, 1995; Otori et al., 1998; Hack et al., 1999; Pang et al., 1999), which means the overstimulation of glutamate receptors could cause the increase of calcium influx and the followed apoptosis and necrosis. We have established the primary retinal cultures (the purity is over 90%) (Li et al., 2016; Wang S. et al., 2017), which is understandable to be used in the next experiments (Petegnief et al., 2001; Oliveira et al., 2011; Miao et al., 2012; Tan et al., 2014; Morgan-Warren et al., 2016).

Recent studies have addressed that CAST/calpain system was involved in cell death following glutamate excitotoxicity (Miao et al., 2012; Curcio et al., 2015; Yan et al., 2016). Calpains are calcium-dependent cysteine proteases, which ubiquitously exist in organisms (Curcio et al., 2016; Machado et al., 2017). The activity of calpains are physiologically regulated by its specific endogenous inhibitor, calpastatin (CAST) (Averna et al., 2001; Volbracht et al., 2005). The CAST/calpain system has drawn more attention since it was implicated in a variety of calcium-related cellular processes, such as survival, proliferation, differentiation, apoptosis, and signal transduction (Senter et al., 1991; Curcio et al., 2016; Martensson et al., 2017). Recent studies have specifically addressed whether the CAST/calpain system plays a role in cell death following glutamate excitotoxicity (Miao et al., 2012; Curcio et al., 2015; Yan et al., 2016). Further, one possible pathway of glutamate excitotoxicity is that activated calpains cleave multiple targets, like apoptosis-induced factor (AIF). Calpain-cleaved AIF (truncated AIF, tAIF) can translocate to the nucleus and provoke DNA degradation, which ultimately results in apoptosis and regulated necrosis (Shang et al., 2014; Curcio et al., 2015; Chen et al., 2016). Although there is evidence for CAST/calpain pathway-dependent cell death, the regulators of this pathway during regulated necrosis is still unclear.

Peptidyl-prolyl *cis/trans* isomerase NIMA-interacting 1 (Pin1), which belongs to the peptidyl-prolyl isomerase family, binds and catalyzes *cis/trans* isomerization of a phosphorylated threonine (T) or serine (S)-proline (P), therefore modulating protein degradation (Liu et al., 2011; Lu et al., 2016; Carnemolla et al., 2017). Pin1 acts as a regulatory molecular, mediating a variety of cellular proteins functions, location, levels, and phosphorylation statuses. It also plays an important role in a number of common cell signaling pathways (Liu, 1979; Kim et al., 2015; Kondo et al., 2015). Accumulating evidence suggest that Pin1 is highly expressed in the CNS and involved in the regulation of neuronal survival, differentiation, and death (Ghosh et al., 2013). Dysregulation of Pin1 has been found in some neuropathological conditions, such as AD, corticobasal degeneration, and retinal diseases (Liu, 1979; Akiyama et al., 2005; Ghosh et al., 2013; Agostoni et al., 2016). Our unpublished preliminary microarray data also demonstrated that the mRNA expression level of *Pin1* in acute retinal ischemia/reperfusion model was sevenfold higher than in the normal rat retina. Further, recent discoveries indicated that Pin1 plays a regulatory role in calpain-induced neuronal death, during glutamate excitotoxicity (Liu et al., 2009; Baik et al., 2015). Another study reported that Pin1 may act with p(T/S)-P in CAST and regulate the function of CAST in endothelial cells (Liu et al., 2011). These reports raised the question of whether Pin1 can interact with CAST and regulate CAST/calpain2 downstream under excessive glutamate condition, and subsequently lead to regulated necrosis in retinal neurons.

To investigate the above questions, we evaluated the effect of Pin1 on CAST/calpain2-modulated necrosis in primary retinal neurons and a RGC-5 cell line under the excessive glutamate condition. Additionally, we performed *in vivo* studies to verify the role of Pin1-CAST/calpain2 pathway in cell necrosis in retinal ganglion cell layer (GCL) and inner nuclear layer (INL) following glutamate excitation. Our work aims to investigate the involvement of the Pin1-CAST/calpain2 pathway in glutamate-mediated excitotoxicity. We expect that the results will provide better understanding and rational interventional targets for neuronal regulated necrosis in the future.

## Materials and Methods

### Primary Retinal Neuron Cultures and *in Vitro* Model Preparation

All experimental procedures used in the present study were approved by the Ethics Committee of the 3rd Xiangya Hospital of Central South University in accordance with the National Institutes of Health (NIH) Guidelines for the Care and Use of Laboratory Animals. Primary retinal neuronal cultures were prepared from 1-day-old neonatal Sprague-Dawley (SD) rat as described previously (Li et al., 2016; Morgan-Warren et al., 2016; Wang S. et al., 2017). In brief, the eyes were removed aseptically, and then the corneas, crystalline lens, and pigment epithelium layer were removed, leaving the retinae. The retinae were placed in Dulbecco’s modified Eagle’s medium (DMEM, GE Healthcare, Logan, UT, United States) containing 0.02% papain and digested in a 5% CO_2_ incubator at 37°C for 10 min. The tissue was then triturated 20 times with a Pasteur pipette and filtered with a 70-mm nylon cell strainer. The cells were counted and plated at a density of 6 × 10^5^ cells/mL. Cells were cultured in 5% CO_2_ incubator at 37°C. Four hours after plating, the medium was replaced with neurobasal medium (Thermo Scientific, Waltham, MA, United States) supplemented with B27 (Thermo Scientific). Culture media were changed every 2 days. On the 7th day, the cultures were treated with 50 μM glutamate (Sinopharm, Beijing, China) for 2 h, and then allowed to recover for 0, 2, 4, 6, 12, or 24 h (Aihara et al., 2014; Kim et al., 2016).

### RGC-5 Cell Line and *in Vitro* Model Preparation

The RGC-5 cell line was provided by the Department of Ophthalmology, Second Hospital of Jilin University in China (Ding et al., 2015). RGC-5 cells were cultured in DMEM (Thermo Scientific) supplemented with 10% FBS (Thermo Scientific) and 1% Penicillin-Streptomycin (Thermo Scientific). The cells were grown in 5% CO_2_ incubator at 37°C. The RGC-5 cells used in the experiment was within 2–3 passages post-thawed. The density of RGC-5 cells was approximately 80% in the T25 flask before the experiment. The RGC-5 cells were treated with 10, 20, 30, 40, or 50 mM glutamate for 1 h (Maher and Hanneken, 2005; Fatma et al., 2008).

### Animal Model *in Vivo* of Glutamate Treatment

The SD rat glutamate model was prepared following the previously reported protocol (Fang et al., 2010). Rats were anesthetized using an intraperitoneal injection with a 1:1 mixed solution (5 mL/kg) of 10% chloralic hydras and 25% urethane. A drop of chloramphenicol eye drop was administered to the left conjunctiva sac. The pupils were dilated with tropicamide and a single dose of 5 μL of 100 mM glutamate in 0.01 M PBS was injected into the vitreous cavity using a 32-gauge Hamilton needle and syringe (Sisk and Kuwabara, 1985). A sham operation was performed as a control. Animals were allowed to survive for 6 h. Each group was composed of four animals.

### Animal Tissue Preparation

At each sacrificed time-point, animals were anesthetized with 10% chloralic hydras (5 mL/kg) and perfused transcardially with 0.9% sodium chloride, followed by 4% paraformaldehyde (PF) in 0.1 M PB. After perfusion, the eyes were enucleated, anterior segments removed, and posterior eyecups were post-fixed in 4% PF overnight at 4°C. Then, the eyes were placed sequentially in 15 and 30% sucrose in 0.1 M PB at 4°C. Next, the eyecups were embedded in Tissue-Tek optimal cutting temperature medium and frozen in liquid nitrogen. After, cryosections were cut, at a thickness of 6 mm, using a microtome (Thermo Scientific). The sections that included the optic nerve were stored at -20°C until use.

### Drug Application

Juglone (i.e., a specific pin1 inhibitor) was dissolved in dimethyl sulfoxide (DMSO, Sigma–Aldrich, St. Louis, MO, United States) as a stock solution. The stock solution was further diluted in culture medium to achieve DMSO concentrations below 0.1%. Juglone was used at a concentration of 10 μM. The drug solution was administered directly to primary retinal neuronal cultures or by intravitreal injection to rats, 2 h before the glutamate treatment.

### siRNA Approach

The siRNA kit against Pin1 was obtained from RIBO-Biology (Guangzhou, China). In companion control experiments, RGC-5 were transfected with the same amount of either a targeting or non-targeting control. Another group with Lipo2000 (Thermo Scientific) alone was used as a normal control. The transfection was performed by following the manufacturer’s instructions. Briefly, the siRNA and transfection reagent were first separately diluted in opti-MEM for 5 min. Then, the diluted siRNA and transfection reagent solutions were mixed together for another 20 min to form the complex. Finally, the cell medium was replaced with opti-MEM, and the transfection complexes were added dropwise to the cultures. The culture medium was replaced with normal medium 6 h after transfection. The cultures were collected for the next experiments 24 h after transfection. Knockdown of calpain2 or Pin1 was validated using western blot.

### Lactate Dehydrogenase (LDH) Release

For *in vitr*o experiments, the LDH cytotoxicity assay kit (Beyotime, Shanghai, China) was used to measure LDH released from necrotic cells into the extracellular space/supernatant upon the rupture of plasma membrane (Xiong et al., 2016; Shang et al., 2017) after the different treatments. Cell-free culture supernatants were collected from 96-well microtiter plate and incubated with the appropriate reagent mixture according to the manufacturer’s instructions at room temperature (RT) for 30 min. For the *in vivo* experiment, the LDH cytotoxicity assay kit was purchased from Jiancheng institutes (Nanjing, China) and used according to the manufacturer’s instructions. Briefly, the retinae were homogenized by sonication in 0.86% NaCl and incubated with the appropriate reagent mixture at 37°C for 30 min. The intensity of the red color that formed in the assay, which was measured at a wavelength of 490 or 450 nm, was proportional to both the LDH activity and percentage of necrotic cells. The percentage of necrotic cell death was calculated by the color intensity of treated cells minus control cells/LDH releasing reagent treated cells minus control cells, from four independent experiments.

### Propidium Iodide (PI) Staining

PI staining was used to identify cells undergoing necrosis (Rosenbaum et al., 2010; Pietkiewicz et al., 2015). Cell cultures on the coverslips were washed twice with PBS and incubated with PI at RT for 20 min in the dark. For animal experiment, 5 μL PI were intravitreally injected, 30 min prior to sacrifice (Huang et al., 2013). Rats were euthanized at the indicated time-point. The eyes were frozen in nitrogen vapor and cryosectioned. The coverslips or retinal sections were washed three times and covered with an anti-fading mounting medium with DAPI (Vector Laboratories, Burlingame, CA, United States). Images were captured using a fluorescence microscope (Olympus, Tokyo, Japan). The percentages of cells were analyzed using Image J software (NIH, Baltimore, MD, United States). The number of PI-positive cells was calculated in every intact captured image.

### Immunofluorescence Staining

Cell cultures on the coverslips were fixed for 20 min with 4% PF and the rat retinal sections were recovered to RT. The coverslips or retinal sections were washed three times for 5 min in ice-cold 0.01 M PBS. Subsequently, the coverslips or retinal sections were blocked for 1 h in blocking buffer, i.e., PBS containing 5% normal bovine serum and 0.3% Triton X-100, and then incubated with combinations of the primary antibodies against the following targets, overnight at 4°C: tAIF (1:500, sc-13116, Santa Cruz), CAST (1:50, sc-2-7799, Santa Cruz, Dallas, TX, United States) and calpain2 (1:100, ab39165, Abcam, Cambridge, United Kingdom). The coverslips or retinal sections were shifted to RT for 30 min, washed three times as described above, and incubated with Alexa-conjugated secondary antibodies (1:200, Jackson Immuno Research, West Grove, PA, United States) for 2 h. After washing three times in PBS, the coverslips or retinal sections were covered with Vectashield mounting medium containing DAPI. The coverslips or retinal sections were stained in parallel and images were acquired, with a fluorescence microscope, using the same settings.

### Real-Time Quantitative Polymerase Chain Reaction (RT-qPCR) Assay

Total RNA was extracted using TRIzol Reagent (Thermo Scientific). For mRNA levels detection, cDNA was synthesized using the Revert Aid First Strand cDNA Synthesis Kit (#K1621) (Fermentas, Canada). RT-qPCR was performed using the Power SYBR Green PCR Master Mix on a MiniOpticon^TM^ Real-Time PCR System (Bio-Rad, United States). The data were analyzed using the 2^-ΔΔ*C_t_*^ method, with CFX Manager Software package (Bio-Rad). GAPDH was used as an internal control.

### Western Blot

For western blot analyses, cell cultures and rat retinal tissues were lysed in RIPA buffer, which contained 1% protease inhibitors (CWBIO, Beijing, China). The extracts were incubated at 4°C for 45 min and then centrifuge at 12,000 *g* for 20 min at 4°C. The supernatant was collected carefully. The protein concentration was determined using a BCA assay and 10 μg of each protein sample was loaded per lane. Proteins were separated using 4–6 or 10% SDS–PAGE gel and transferred to a nitrocellulose membrane (GE Healthcare). The membranes were blocked with blocking buffer containing 5% non-fat milk in Tris-buffered saline with 0.1% Tween 20 for 1 or 3 h, at RT, before being incubated overnight at 4°C in blocking buffer containing the following primary antibodies: CAST (1:200), calpain2 (1:500), Pin1 (1:100, 3722S, Cell Signaling, Danvers, MA, United States), tAIF (1:50), PTEN (1:50, BA1377, Boster, Wuhan, China), GAPDH (1:5,000, AF0006, Beyotime), and β-Actin (1:5,000, AA128, Beyotime). The membranes were shifted to RT for 30 min. After washing three times, the membranes were incubated in blocking buffer containing HRP-conjugated anti-rabbit or anti-mouse IgG (1:2,000, Beyotime) for 2 h, at RT. After washing three times, the immunoreactive bands were visualized by low or high sensitivity chemiluminescence reagent (CWBIO). GAPDH was used as an internal reference control. Integrated density values of specific proteins were quantified using Image J software and normalized to the GAPDH or β-Actin values.

### Phos-tag^TM^ SDS–PAGE

The protocol was similar with Western blot, except for the differences outlined below. The phosphatase inhibitor was added to the tissues prepared for proteins extracts. The phos-tag^TM^ (Wako, 192-18001-EA-S, Richmond, VA, United States) and Mn^2+^ were added to the SDS–PAGE gel at a concentration of 50 and 100 μM individually. Before transferring onto membrane, the gel was washed with transferring buffer containing 1 μM EDTA for 15 min, and then with transferring buffer without EDTA for 15 min.

### Flow Cytometry

The RGC-5 cells were trypsinized, followed by one wash. Cells were then re-suspended in 500 μL of binding buffer, and 5 μL of PI were added. After being incubated for 10 min at RT in the dark, the cells were washed and analyzed using FACSCalibur^TM^ (Becton, Dickinson Company, Franklin Lakes, NJ, United States). The percentages of cells in each quadrant were analyzed using FlowJo software (FlowJo LLC, Ashland, OR, United States). Statistical analyses of flow cytometry results were conducted by calculating the number of PI-positive cells (Yang et al., 2017).

### Simulation of Protein Binding Confirmation

The three dimensional structure of proteins were built based on available protein crystal diffraction structure and frozen electron microscopic structure by homologous modeling (Swiss Model) (Schwede et al., 2003). Protein surface electrostatic potential energy were calculated using APBS (figures were produced using PyMOL) (Baker et al., 2001).

### Co-immunoprecipitation (Co-IP)

Cultured retinal neurons were lysed with non-denaturing lysis buffer, protease and phosphatase inhibitor mixture. Then, the extracted protein was incubated with CAST antibody (1:50, Cell Signaling) or Pin1 (1:100, 10495-1-AP, Proteintech, Wuhan, China) coupled to protein A/G Agarose beads, overnight at 4°C. Finally, the bound proteins were analyzed using western blot.

### Flash Electroretinogram (fERG)

The RM6240 system (Chengdu Instrument Factory, Chengdu, China) was used for fERG recording. After the drug treatment, the rats were dark-adapted for 6 h. Under dim red illumination, the rats were anesthetized using an intraperitoneal injection with a 1:1 mixed solution (5 mL/kg) of 10% chloralic hydras and 25% urethane. Then, a recording electrode was inserted into the anterior chamber. The reference and ground electrodes were placed on the subcutaneous layer of the forehead and tail base, respectively. A bandpass filter of 10 Hz was used and the lash luminance was 1.6 cd/s/m^2^. Each eye was exposed to flashes, three times, at a 5-min interval. When one eye was recorded, the contralateral eye was covered. All procedures were repeated at least four times. The amplitude of b wave was calculated from the bottom of a wave to the peak of b wave.

### Statistical Analysis

Figure panels were assembled using Photoshop CC (Adobe Systems Incorporated, San Jose, CA, United States). The measurement data were presented as the mean ± standard deviation (SD). One-way analysis of variance and independent sample *t*-tests were used to analyze the data, with GraphPad Prism 5 software (GraphPad Software, Inc., San Diego, CA, United States). Statistical significance was set at *p* < 0.05.

## Results

### Glutamate Induces Necrosis in Primary Retinal Neurons

**FIGURE 1:**
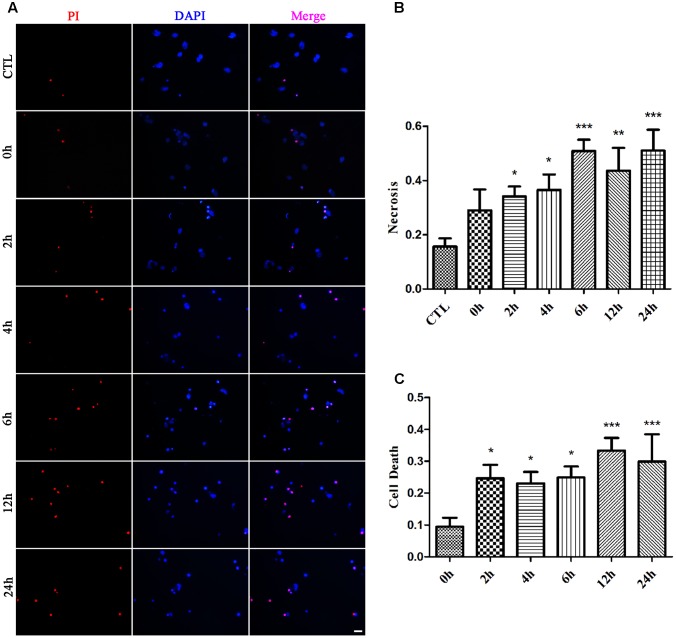
Necrotic retinal neurons determined by propidium iodide (PI) staining and lactate dehydrogenase (LDH) release after glutamate treatment. **(A)** Glutamate treated retinal neurons were stained with PI (red). Nuclei were counterstained with DAPI (blue). **(B)** Quantitative analysis of PI stained neurons after glutamate treatment. **(C)** The percentage of necrotic neurons were determined by LDH release. Data are expressed as means ± SD (*n* = 5, each). ^∗^*p* < 0.05, ^∗∗^*p* < 0.01, ^∗∗∗^*p* < 0.001 vs. respective CTL or 0 h group. Scale bar: 20 μm in all panels.

We first examined whether necrosis occurred in primary retinal neurons following glutamate treatment. In these experiments, cultured retinal neurons were treated with 50 μM glutamate for 0, 2, 4, 6, 12, or 24 h, respectively. At our observation time-points, the number of PI-positive cells gradually increased from 2 h and peaked at 6 h following glutamate treatment (**Figures 1A,B**). We also conducted the LDH cytotoxicity assay to evaluate the percentage of necrosis cells. When compared with 0 h, the increased percentage of necrosis cells were significant at all observed time-points (**Figure 1C**). These results demonstrated a glutamate-dependent increase in necrosis in neuronal retinal primary cultures.

### Effect of Glutamate on Pin1-CAST/Calpain2 Pathway

**FIGURE 2:**
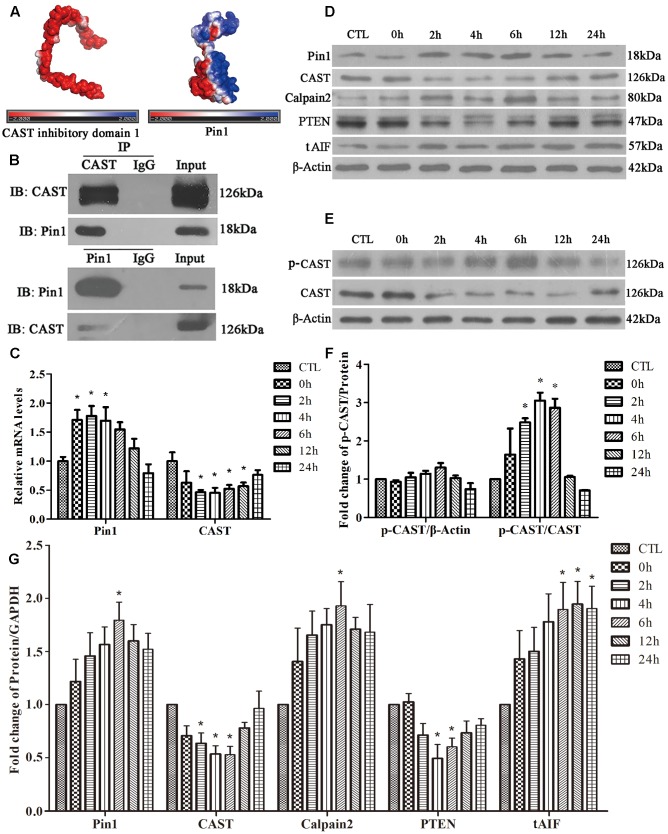
Effect of glutamate on Pin1-CAST/calpain2 pathway and the interaction of Pin1 with CAST. **(A)** Protein surface electrostatic potential energy were calculated using APBS (figures were produced by using PyMOL). **(B)** IP assay of CAST and Pin1. **(C)** *Pin1* and *CAST* mRNA levels in retinal neurons following glutamate treatment. Data are expressed as means ± SD (*n* = 4, each). ^∗^*p* < 0.05 vs. CTL group. **(D)** Pin1, CAST, calpain2, PTEN, and tAIF expression in retinal neurons following glutamate treatment. **(E)** P-CAST and CAST levels in retinal neurons following glutamate treatment. **(F)** The statistical analysis of p-CAST/CAST and p-CAST/β-Actin in retinal neurons following glutamate treatment. Data are expressed as means ± SD (*n* = 4, each). ^∗^*p* < 0.05 vs. CTL group. **(G)** The statistical analysis of Pin1, CAST, calpain2, PTEN, and tAIF expression in retinal neurons following glutamate treatment. Data are expressed as means ± SD (*n* = 5, each). ^∗^*p* < 0.05 vs. CTL group.

Possible interaction between Pin1 and CAST was investigated first using a computer. The CAST inhibitory domain 1 (the whole structure data of CAST were not showed) had negative electrostatic potential on most of its surface (**Figure 2A**). Pin1, however, displayed positive electrostatic potential, which could be properly docked to the CAST (**Figure 2A**). The four inhibitory domains of CAST were similar to previous reports. Therefore, all CAST inhibitory domains had the potential to bind Pin1 by electrostatic interaction. Further, we performed Co-IP, which showed that Pin1 also interacted with endogenous CAST (**Figure 2B**).

Next, RT-qPCR was performed to analyze the changes of *Pin1* and *CAST* mRNA levels. As shown in **Figure 2C**, glutamate significantly upregulated the level of *Pin1* mRNA at 0, 2, or 4 h, but downregulated the *CAST* mRNA level at 2, 4, 6, 12, or 24 h (**Figure 2C**). Western blot was used to detect the proteins expression. We first showed one complete blot of each antibody used in **Supplementary Figure S1** to evaluate specific binding of antibodies to proteins. Western blot analysis further showed that Pin1 expression increased after 4, 6, 12, or 24 h of glutamate treatment (**Figures 2D,G**). The expression of CAST, however, decreased after 2, 4, or 6 h of glutamate treatment (**Figures 2D,G**). Western blot results further demonstrated that the calpain2 expression levels at 4, 6, 12, or 24 h were increased after glutamate treatment (**Figures 2D,G**). The tAIF (a calpains cleave target) protein levels demonstrated a similar upregulation with calpain2 at the different time-points (**Figures 2D,G**). In addition, the level of PTEN, which is regulated by specific proteolysis of calpain2, was used to represent the degree and duration of calpain2 activation. The increased expression of calpain2 was also associated with the activation of calpain2, as indicated by the decrease in the expression levels of PTEN (**Figures 2D,G**). These results showed that glutamate treatment could increase Pin1 and calpain2 activity, but decrease CAST expression in retinal neuronal cultures.

It is well-known that phosphorylation of the N-terminal of CAST L-domain reduces its interaction with calpain (Salamino et al., 1997; Liu et al., 2011). Next, using phos-tag SDS–PAGE, we assessed whether the phosphorylation of CAST changed after glutamate treatment. Interestingly, the levels of phosphorylated CAST (p-CAST) were almost same at every time-point (**Figures 2E,F**). However, the relative p-CAST compared with total CAST increased at 2, 4, and 6 h after glutamate treatment (**Figures 2E,F**). This finding suggested that the level of CAST phosphorylation was enhanced after the glutamate treatment in retinal neuronal cultures.

### Pin1 Regulates CAST/Calpain2 Pathway via CAST Transcription, Expression, and Phosphorylation

**FIGURE 3:**
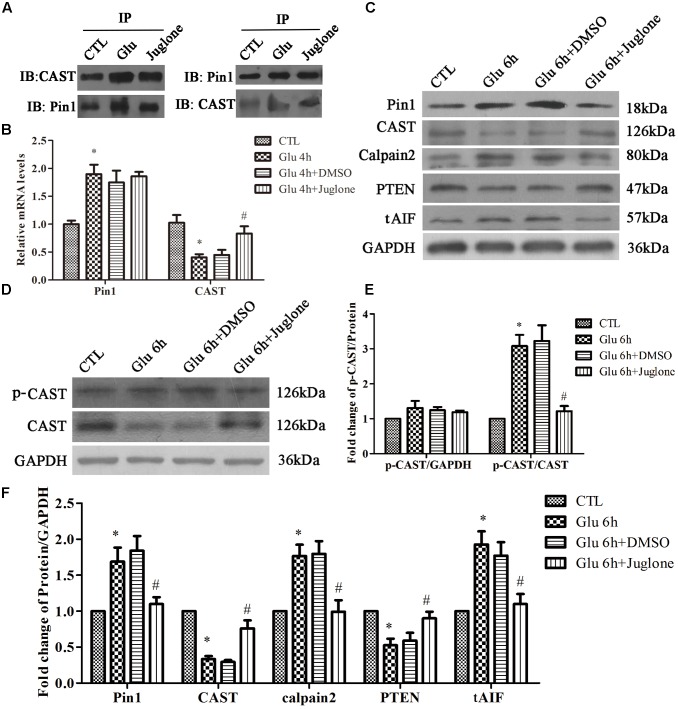
Pin1 regulates CAST/calpain2 pathway. **(A)** IP assay of CAST and Pin1 after glutamate and pretreated with juglone. **(B)** *Pin1* and *CAST* mRNA levels in retinal neurons following glutamate and pretreated with juglone before glutamate treatment. Data are expressed as means ± SD (*n* = 4, each). ^∗^*p* < 0.05 vs. CTL group, ^#^*p* < 0.05 vs. Glu 4 h group. **(C)** Pin1, CAST, calpain2, PTEN, and tAIF expression in retinal neurons following glutamate and pretreated with juglone before glutamate treatment. **(D)** P-CAST and CAST expression in retinal neurons following glutamate and pretreated with juglone before glutamate treatment. **(E)** The statistical analysis of p-CAST/CAST and p-CAST/β-Actin in retinal neurons following glutamate and pretreated with juglone before glutamate treatment. Data are expressed as means ± SD (*n* = 4, each). ^∗^*p* < 0.05 vs. CTL group, ^#^*p* < 0.05 vs. Glu 6 h group. **(F)** The statistical analysis of Pin1, CAST, calpain2, PTEN, and tAIF expression in retinal neurons following glutamate treatment. Data are expressed as means ± SD (*n* = 5, each). ^∗^*p* < 0.05 vs. CTL group, ^#^*p* < 0.05 vs. Glu 6 h group.

To examine the role of Pin1 in CAST/calpain2 pathway after glutamate treatment, we investigated the effect of juglone, which can covalently inactivate and degrade Pin1 (Baik et al., 2015). The Co-IP results (**Figure 3A**) showed that the interaction between Pin1 and CAST was increased in the glutamate groups compared with control groups. Additionally, the interaction between the Pin1 and CAST could be decreased through pretreatment with juglone (**Figure 3A**). In the RT-qPCR experiments, we chose the 4-h glutamate time-point as our primary intervention time-point, since the changes of *Pin1* and *CAST* mRNA were significant compared with CTL group. Compared with the 4-h glutamate groups, juglone treatment did not change the mRNA levels of *Pin1* (**Figure 3B**). Juglone, however, did decrease the protein levels of Pin1, when compared with the 6-h glutamate group. Compared with that in the CTL group, the Pin1 and CAST protein levels were significantly changed at this time-point (**Figures 3C,F**). This indicated that although juglone may degrade Pin1 directly, it has no effect on the transcription of Pin1 (Baik et al., 2015). Meanwhile, after Pin1 inactivation and degradation by juglone, the decreased the levels of *CAST* mRNA and protein induced by glutamate was almost recovered, as shown by RT-qPCR and western blot results (**Figures 3B,C,F**). Moreover, the juglone treatment decreased the expression of calpain2 and tAIF compared with that in the glutamate group (**Figures 3C,F**). In addition, the expression of PTEN was nearly recovered in the juglone-treatment group (**Figures 3C,F**). These results indicated that Pin1 inhibition by juglone may regulate the CAST/calpain2 pathway through the upregulation of CAST transcription and expression.

Next, we assessed the change of CAST phosphorylation after Pin1 inhibition. The level of p-CAST was unchanged after juglone application (**Figures 3D,E**). However, relative p-CAST compared with total CAST in the juglone group was decreased than in the glutamate group (**Figures 3D,E**). These results indicated that the phosphorylation effects were blocked by the inhibition of Pin1 due to the juglone application.

**FIGURE 4:**
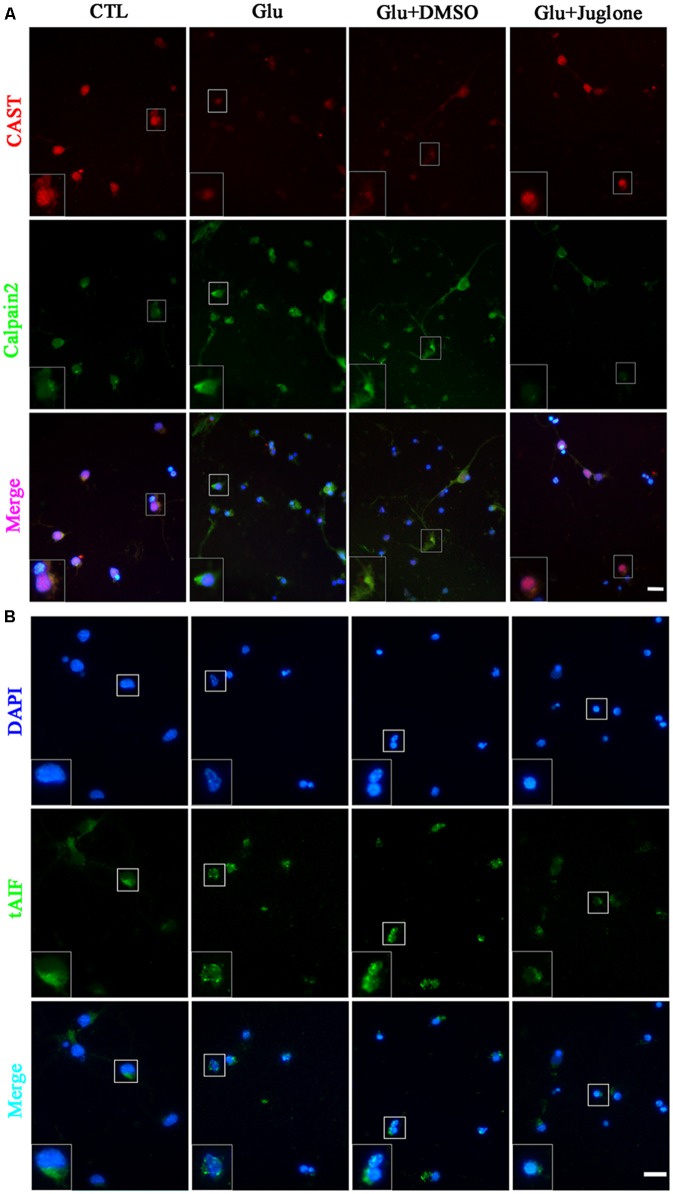
Immunofluorescence staining **(A)** Immunofluorescence staining of CAST and calpain2 following juglone pretreatment, before the 6-h glutamate treatment. **(B)** Immunofluorescence staining of tAIF and DAPI following juglone pretreatment, before the 6-h glutamate treatment. Scale bar: 20 μm. Selected enlarged area shown inset, scale bar: 10 μm.

Double immunofluorescence was used to further evaluate the regulatory role of Pin1 on CAST/calpain2. The immunostaining results showed that CAST was predominantly located in the cytoplasm and nucleus, and slightly distributed in the dendrite (**Figure 4A**). The immunofluorescence intensity of CAST showed a large decrease in the cytoplasm, nucleus, and dendrites after glutamate treatment (**Figure 4A**). However, the decrease in CAST was almost recovered after the juglone application (**Figure 4A**). Calpain2 immunostaining was mainly present in the cytoplasm and nucleus. Calpain2 expression was increased within both the cytoplasm and the nucleus after the 6-h glutamate treatment (**Figure 4A**). Calpain2 immunostaining in the dendrites of neurons was also slightly enhanced after 6 h. The increase in the level of calpain2, however, was decreased after the juglone application (**Figure 4A**). A dramatic increase of tAIF immunofluorescence was also observed at 6 h after glutamate treatment. Furhtermore, the tAIF translocated into the nucleus showed by double labeling with DAPI (**Figure 4B**). After the juglone application, the nuclear translocation and enhanced immunofluorescence intensity of tAIF, induced by glutamate, were no longer observed (**Figure 4B**).

### The Role of Pin1 in Regulated Necrosis

**FIGURE 5:**
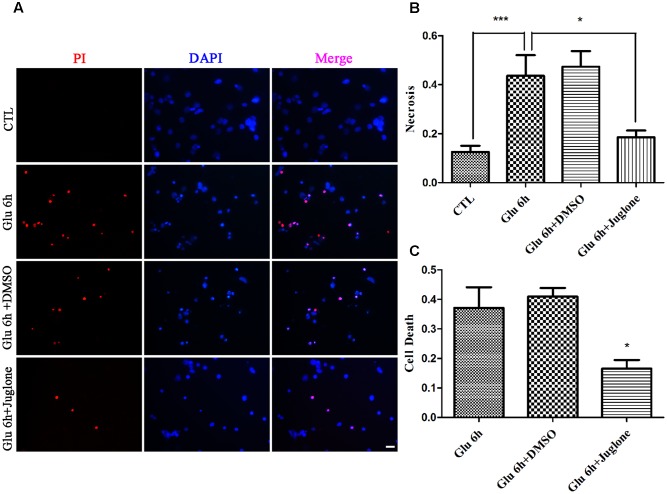
Necrosis determined by propidium iodide (PI) staining and LDH release. **(A)** Retinal necrotic neurons were stained with PI (red). Nuclei were counterstained with DAPI (blue). **(B)** Quantitative analysis of PI-stained retinal neurons. Data are expressed as means ± SD (*n* = 5, each). ^∗∗∗^*p* < 0.001, ^∗^*p* < 0.05. **(C)** The percentage of necrotic neurons were determined by LDH release. Data are expressed as means ± SD (*n* = 5, each). ^∗^*p* < 0.05 vs. Glu 6 h group. Scale bar: 20 μm in all panels.

We evaluated the effect of juglone-induced Pin1 inhibition on retinal neuronal necrosis. As shown in PI staining and LDH assay, there was a reduction in necrosis in juglone-treated retinal neurons, following the 6-h glutamate treatment (**Figures 5A–C**). These results indicated that inhibition of Pin1 by juglone application can decrease the necrosis following glutamate treatment in retinal neurons.

### Pin1 Modulates Regulated Necrosis via the CAST/Calpain2 Pathway in the RGC-5 Cell Line

After initial investigation of primary cultured retinal neurons treated with Pin1 inhibitors following glutamate treatment, we used siRNA to investigate the regulatory role of Pin1 in glutamate-induced necrosis in the RGC-5 cell line. RGC-5 cells were subjected to different concentrations (10, 20, 30, 40, or 50 mM) of glutamate for 1 h. There was a gradient increase in the number of necrotic cells with increasing concentrations of glutamate (Data not shown); the 40 mM glutamate concentration was chosen for subsequent experiments. In this section, we used glutamate concentrations in the millimolar range to induce necrosis in RGC-5, whereas a micromolar concentration range was used for primary cultures because RGC-5 requires higher glutamate concentrations to display similar features to primary neurons (Maher and Hanneken, 2005; Fatma et al., 2008).

**FIGURE 6:**
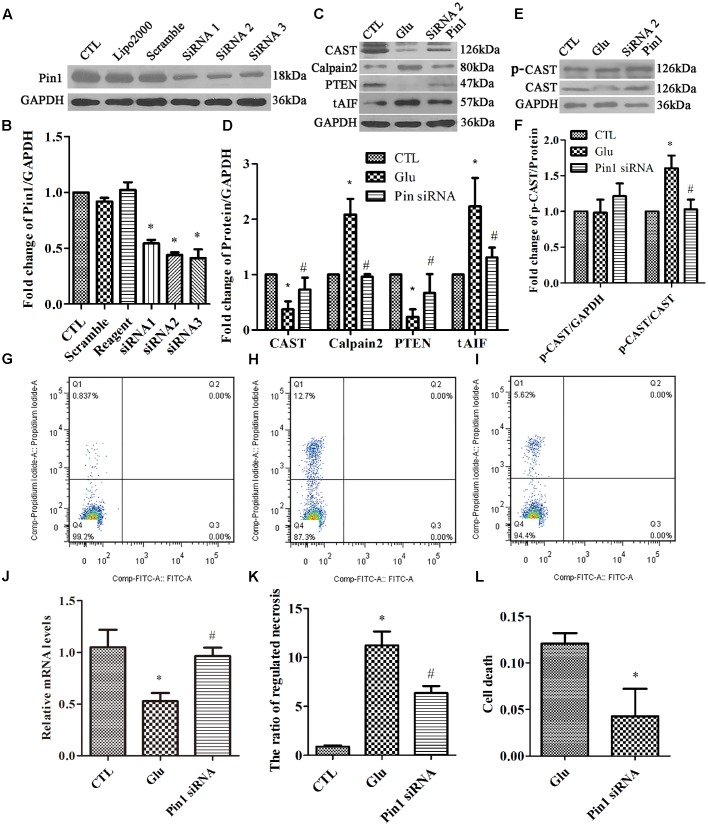
Expression of Pin1, CAST, calpain2, PTEN, and tAIF, and quantification of necrosis in RGC-5 cells pretreated with siRNA before the glutamate treatment. **(A)** Pin1 expression in RGC-5 cells after siRNA knockdown. **(B)** Statistical analysis of Pin1 expression in RGC-5 cells after siRNA knockdown. Data are expressed as means ± SD (*n* = 3, each). ^∗^*p* < 0.05 vs. CTL group. **(C)** CAST, calpain2, PTEN, and tAIF expression in RGC-5 cells pretreated with *Pin1* siRNA before the glutamate treatment. **(D)** Statistical analysis of CAST, calpain2, PTEN, and tAIF levels in RGC-5 cells following glutamate and pretreated with *Pin1* siRNA before the glutamate treatment. Data are expressed as means ± SD (*n* = 4, each). ^∗^*p* < 0.05 vs. CTL group, ^#^*p* < 0.05 vs. Glu group. **(E)** p-CAST and CAST expression in RGC-5 cells pretreated with *Pin1* siRNA before the glutamate treatment. **(F)** Statistical analysis of p-CAST/CAST and p-CAST/β-Actin levels in RGC-5 cells following glutamate treatment and *Pin1* siRNA pretreatment before glutamate treatment. Data are expressed as means ± SD (*n* = 4, each). ^∗^*p* < 0.05 vs. CTL group, ^#^*p* < 0.05 vs. Glu group. **(G)** Flow cytometry results for necrotic cells in the CTL group. **(H)** Flow cytometry results of necrotic cells in the Glu group. **(I)** Flow cytometry results of necrotic cells in *Pin1* siRNA group. **(J)** *CAST* mRNA levels in RGC-5 cells following glutamate treatment and *Pin1* siRNA pretreatment prior to the glutamate treatment. Data are expressed as means ± SD (*n* = 4, each). ^∗^*p* < 0.05 vs. CTL group, ^#^*p* < 0.05 vs. Glu group. **(K)** Statistical analysis of flow cytometry results. Data are expressed as means ± SD (*n* = 4, each). ^∗^*p* < 0.05 vs. CTL group, ^#^*p* < 0.05 vs. Glu group. **(L)** Statistical analysis of LDH detection. Data are expressed as means ± SD (*n* = 4, each). ^∗^*p* < 0.05 vs. Glu group.

In our present study, we investigated whether Pin1 knockdown can reduce the necrotic cells during glutamate excitotoxicity. RGC-5 cells were transfected with *Pin1* siRNA for 24 h. Scrambled siRNA and transfection-reagent treated cells were compared. The western blot results showed a significant reduction in Pin1 protein levels in RGC-5 cells treated with *Pin1* siRNA (**Figures 6A,B**). Western blot results further indicated that, cells subjected to *Pin1* siRNA had an augmented decrease in CAST and PTEN levels, while the increase in calpain2 and AIF levels was reduced (**Figures 6C,D**). Meanwhile, RT-qPCR demonstrated that CAST was reduced after the glutamate treatment. Further, the decrease in CAST was augmented after *Pin1* siRNA application (**Figure 6J**). The level of p-CAST, however, remained unchanged after glutamate and *Pin1* siRNA application. The relative level of p-CAST compared with total CAST in the siRNA group was lower than in the glutamate treatment group (**Figures 6E,F**). This further indicated that CAST is further downstream of Pin1 and that the phosphorylation of CAST is modulated by Pin1.

Finally, flow cytometry (**Figures 6G–I**) and LDH assay (**Figure 6L**) were used to study cell necrosis. Statistical analysis of both flow cytometry and LDH detection (**Figures 6K,L**) results demonstrated that the ratio of RGC-5 cell necrosis increased when exposed to glutamate (**Figure 6H**), than in the control group (**Figure 6G**). Further, the percentage of necrotic cells was decreased after the *Pin1* siRNA pretreatment (**Figure 6I**).

Taken together, this study provided further evidence that Pin1 plays a role in glutamate injury in retinal neurons and that Pin1 may be an upstream regulatory molecular of the CAST/calpain pathway.

### Pin1 Modulates Regulated Necrosis via the CAST/Calpain2 Pathway in Retinal GCL and INL *in Vivo*

**FIGURE 7:**
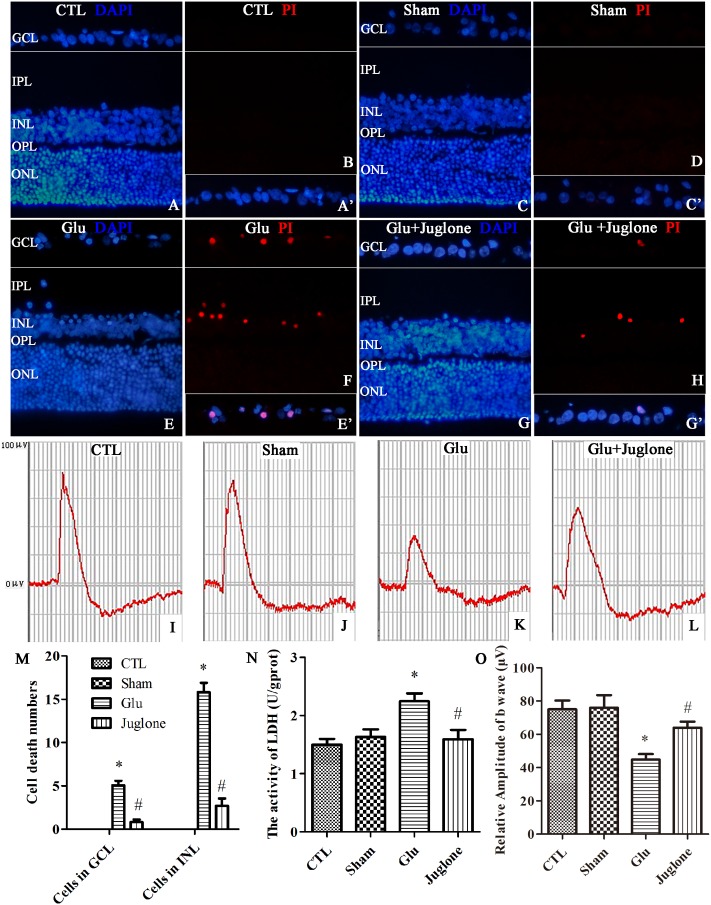
Necrosis was determined by propidium iodide (PI) staining and LDH release, while visual function was detected using flash electroretinogram (fERG) in retina after glutamate treatment *in vivo.* **(A,C,E,G)** Nuclei were stained with DAPI (blue). **(B,D,F,H)** Retinal necrotic neurons were stained with PI (red). **(A′,C′,E′,G′)** The framed areas show merged nuclei and PI staining in the GCL as indicated. **(I–L)** Representative fERG results. **(M)** Quantitative analysis of PI-stained cells in GCL and INL in retina, Data are expressed as means ± SD (*n* = 4, each). ^∗^*p* < 0.05 vs. CTL group, ^#^*p* < 0.05 vs. Glu group. **(N)** The percentage of necrosis cells were determined by LDH release, Data are expressed as means ± SD (*n* = 4, each). ^∗^*p* < 0.05 vs. CTL group, ^#^*p* < 0.05 vs. Glu group. **(O)** Quantitative analysis of the b-wave amplitudes. Data are expressed as means ± SD (*n* = 4, each). ^∗^*p* < 0.05 vs. CTL group, ^#^*p* < 0.05 vs. Glu group. GCL, ganglion cell layer; IPL, inner plexiform layer; INL, inner nuclear layer; OPL, outer plexiform layer; ONL, outer nuclear layer. Scale bar: 20 μm in all panels.

First, PI were intravitreally injected to examine necrotic cells in the rat retina. Double immunofluorescence of DAPI and PI showed an increased in the PI-positive necrotic cells in the GCL and INL in the glutamate groups (**Figures 7E,F**) than that in the control (**Figures 7A,B**) and sham groups (**Figures 7C,D**). Then, we investigated the role of juglone in glutamate-induced necrosis *in vivo*. PI staining results demonstrated a significant reduction in necrotic cells with juglone pretreatment (**Figures 7G,H**). **Figure 7M** shows quantitative analysis of PI-stained cells in the GCL and INL. An *in vivo* LDH cytotoxicity assay was also conducted. Compared with the control and sham groups, LDH release was increased in the glutamate group (**Figure 7N**). However, there was a decrease in the increased LDH release in the juglone pretreatment group (**Figure 7N**). Taken together, these results indicated that juglone could provide a protective role to retinal cells in the GCL and INL against glutamate-induced necrosis.

We then performed fERG to evaluate visual function (Piano et al., 2016). The fERG results showed a significant decrease in the amplitudes of b-wave in the glutamate group than in the control and sham groups (**Figures 7I–K**). However, the amplitudes of b-wave were all enhanced in the juglone-treated group than in the glutamate group (**Figure 7L**). **Figure 7O** shows the quantitative analysis of the b-wave amplitudes. These results demonstrated that the protective effects of juglone on visual function following glutamate excitotoxicity.

Together these results suggested that Pin1 plays an important role in glutamate-induced necrosis and dysfunction *in vivo*.

## Discussion

In the current study, we investigated the role of Pin1 in CAST/calpain2 pathway-modulated regulated necrosis, which was induced by glutamate, in rat retinal neurons, RGC-5 cell line, and *in vivo*. Necrosis occurred in primary cultured retinal neurons exposed to glutamate. We further demonstrated that Pin1 plays a role in CAST/calpain2-mediated regulated necrosis. Then, we used the RGC-5 cell line combined with siRNA approach to verify the roles of Pin1 in the CAST/calpain2-mediated regulated necrosis. Finally, using an animal model, we also demonstrated the neurodegenerative role of Pin1 overexpression *in vivo* in glutamate-induced regulated necrosis in the rat retina. These findings suggested that the involvement of the Pin1-CAST/calpain2 pathway in glutamate-mediated excitotoxicity. This finding will provide a potential target to protect neurons from necrosis, in neurodegenerative diseases, such as glaucoma, diabetic retinopathy, and even CNS diseases.

Recently, regulated necrosis has been proposed as a form of programed cell death, which is distinctive from apoptosis (Pasparakis and Vandenabeele, 2015). Regulated necrosis plays a crucial role in various physiological processes (Galluzzi et al., 2014). Dysregulation of regulated necrosis contributes to the development of diseases, such as cancer, and neurodegeneration (Galluzzi et al., 2014; Pasparakis and Vandenabeele, 2015). Although regulated necrosis includes several cell death types, such as, receptor interacting protein 3-dependent necroptosis that can be triggered by tumor necrosis factor-α (Degterev et al., 2008; Jiang et al., 2014), erastin-induced ferroptosis that requires iron, mitochondrial permeability transition dependent regulated necrosis, etc. (Gao et al., 2015; Kong et al., 2017). These regulated types of necrosis do not have completely independent signaling pathways, but exhibit some degree of crosstalk (Galluzzi et al., 2014; Zhao et al., 2015). Many reports demonstrated that glutamate, the most widely distributed excitatory amino acid in CNS, could activate membrane receptors and lead to an influx of calcium to cells, which eventually results in regulated necrosis (Lalkovicova and Danielisova, 2016; Ozaki et al., 2016).

The three glutamate subtypes, i.e., NMDAR, AMPAR, and KAR are all expressed in the retinal neurons (Fang et al., 2010). Some reports demonstrated that glutamate-induced cell death of young rat retinal neurons is primarily mediated by AMPAR/KAR, which are calcium-permeable (Otori et al., 1998; Luo et al., 2001). Conversely, others demonstrated cultured neonatal rat retinal neurons are susceptible to NMDAR-induced cell death (Pang et al., 1999; Fang et al., 2010). Although distinct roles of the three glutamate receptors involved in retinal neuronal death have not been clarified in the present study, neurotoxicity-mediated by the overstimulation of glutamate receptors caused an increased calcium influx, which was followed apoptosis and necrosis. Thus, in our study, we mainly focused on calcium overload-induced regulated necrosis after glutamate treatment.

Calpains are calcium-dependent cysteine proteases, which ubiquitously exist in organisms and are widely considered to be important mediators in neurodegeneration that is induced by calcium overload (Sanges et al., 2006). There are two main calpain isoforms in the CNS, i.e., calpain1 and calpain2. Our unpublished preliminary data demonstrated the increase in calpain2 activity while the activity of calpain1 was inhibited in primary retinal neurons after glutamate treatment. This discrepancy could be attributed to different responses and roles of calpain1 and calpain2 to excitotoxicity, as activated calpain1 is neuroprotective, but activated calpain2 is neurodegenerative, under excessive neurotoxic condition (Wang et al., 2014, 2016). Another reason may be that calpain1 and calpain2 require μM and mM levels of calcium, respectively, for activation *in vitro* (Averna et al., 2007). In the current study, it was conceivable that the protective role of calpain1 may be inhibited, while the calpain2 activity may be enhanced by excessive glutamate. Given the different roles and changes of calpain1 and calpain2, we focused on the activated calpain2-mediated retinal neuronal regulated necrosis after glutamate injury. Further investigation is warranted to clearly define the regulation and function of calpain1 in regulated necrosis.

Calpain activity is tightly regulated by CAST gene transcription and protein expression (Blomgren et al., 1999; Sizova et al., 2014). Our study showed that the increase in calpain2 expression and activity was correlated with the decrease in CAST transcription and expression after glutamate treatment. In addition, CAST can be activated by exposure to phosphoprotein phosphatases. Phosphorylation of active CAST by protein kinases promotes a decrease in its inhibitory efficiency on calpain (Salamino et al., 1997; Liu et al., 2011). In our experimental results, the phosphorylation level of CAST was not changed by the glutamate treatment. The relative p-CAST level compared with the total CAST level, however, was increased. This indicated that increased p-CAST may decrease its inhibitory effect on calpain2, leading to subsequent activation of calpain2. These results corroborated with previous studies demonstrating that the loss of the inhibitory effect of CAST inhibitory on calpain activity will led to neurodegeneration.

Reversible phosphorylation of (T/S)-P is an important regulatory switch in controlling cellular processes (Ghosh et al., 2013). Pin1 has a two-domain architecture consisting of a N-terminal WW domain and a C-terminal PPIase domain (Liu et al., 2011; Lu et al., 2016; Carnemolla et al., 2017). The WW domain of Pin1 specifically recognizes p(T/S)-P of proteins and catalyzes the *cis/trans* conformational change of the peptide bond (Ghosh et al., 2013). As reported, Pin1 interacted with many membrane receptors and signaling proteins that are closely associated with neuronal cell death, such as apoptotic and necrotic signaling pathways. For example, increased levels of Pin1 mRNA and protein may be an important neurotoxic event in the process of Parkinson disease (PD) (Ghosh et al., 2013). Upregulated Pin1 may contribute to the phosphorylation of α-synuclein aggregation via modulating kinase and phosphatase activity (Ghosh et al., 2013). As a result, Pin1 knockdown or pretreatment with juglone, i.e., a Pin1 specific inhibitor, could provide protective roles against PD (Tang et al., 2017). Besides, Pin1 has been reported to play a regulated role in neuronal death during glutamate excitotoxicity and influx of calcium (Liu et al., 2009; Baik et al., 2015). Our study also found that both mRNA and protein levels of Pin1 were increased after glutamate treatment, which were consistent with others reports that Pin1 can be regulated transcriptionally and translationally (Lu and Hunter, 2014). These results indicated that Pin1 may sever as a sensor of calcium in some diseases (Sacchi et al., 2017). It is widely known that Pin1 is a direct target of E2 transcription factor 1 (E2F1) and the activation of E2F1 is critical for Pin1 promoter activity (Lu and Zhou, 2007; Lu and Hunter, 2014). In the neurodegenerative process, over-activation of E2F1 lead to the increased activity of Pin1 promoter (Lu and Zhou, 2007). These reports suggest that Pin1 may be subject to E2F1-mediated transcriptional regulation in response to increased calcium induced by glutamate. In the previous research, Pin1 can be degraded by an ubiquitin-mediated pathway (Wulf et al., 2005). The phosphorylated on Ser^16^ and Ser^65^ of Pin1 also can reduce Pin1’s ubiquitylation (Kesavapany et al., 2007; Lu and Zhou, 2007; Sacktor, 2010). These reports suggest that glutamate may increase phosphorylation at Ser^16^ and Ser^65^ responding to increased calcium influx and aberrantly activate Pin1 by decreased ubiquitylation (Kesavapany et al., 2007).

Recent studies indicated that Pin1 plays a regulatory role in neuronal death induced by calpain during glutamate excitotoxicity (Liu et al., 2009; Baik et al., 2015). Little is known about the relationship between Pin1 and calpain (Galas et al., 2006). Since CAST has several (S/T)-P sites and can be phosphorylated on these sites in different cell types, it is possible that Pin1 may interact with CAST and regulate calpain activity as Pin1 could bind the p(S/T)-P site (Adachi et al., 1991; Liu et al., 2011). Consistent with this hypothesis, research demonstrated that Pin1 binds and affects CAST in endothelial cells (Liu et al., 2011). The current study is the first to show that CAST can interact with Pin1 in retinal neurons, using Co-IP and confirming protein binding using a computer simulation. Besides, our Co-IP results further showed that, following glutamate injury, the binding between Pin1 and CAST was increased compared with control groups. After application of juglone, the interaction was decreased. These results indicated that the interaction between Pin1 and CAST could be an important event for evaluating the progress of necrosis. In addition, some research indicated that Pin1 interacts with many cell membrane receptors, like NMDA receptors (Averna et al., 2015b; Tang et al., 2017), which are also associated with calpain as previously reported (Averna et al., 2015a; Martines et al., 2017). This suggests that Pin1, CAST, and calpain might form a complex that can combine to NMDA receptors.

To further verify that the CAST/calpain2 pathway was downstream of Pin1, we analyzed changes in activity of CAST and calpain2 after inhibition of Pin1 to juglone and siRNA. From our data, juglone, which might inactivate Pin1 by modifying sulfhydryl groups and degrade Pin1 by the proteasome (Chao et al., 2001), is effective for Pin1 inhibition as observed by the decrease in the expression of Pin1. However, the juglone treatment did not change the mRNA levels of *Pin1*. Thus, although juglone may not affect Pin1 transcription, it can degrade the Pin1 protein (Ghosh et al., 2013). Decreased Pin1 expression, induced by siRNA or juglone, significantly enhanced CAST mRNA and protein levels, decreased relative p-CAST levels, which in turn enhanced the inhibitory efficiency on calpain2. This consequently decreased the number of necrotic cells and rescued the impaired visual function, which was induced by glutamate, *in vivo*. It is known that the WW domain of Pin1 may interact with p(S/T)-P sites. These provide the basis that interaction between p(S/T)-P site of CAST and WW domain of Pin1 (Liu et al., 2011). However, the regulatory mechanism of Pin1 affects transcription and translation of CAST is still need to explore. Pin1 has been found in regulating transcriptional activity of several molecules by modulating theirs nuclear activity (Rustighi et al., 2009). Like under DNA damage condition, the mRNA level and activity of p53 were decreased mediated by Pin1 (Zheng et al., 2002). The reason may be that Pin1 can modulate the DNA-binding activity and transcriptional activity by changing the conformation of p53 (Zheng et al., 2002). Thus, in the present research, our results suggest that increased expression of Pin1 induced by glutamate results in the decreased transcription CAST through compromised DNA-binding activity. Pin1 also has been reported to work with different E3 ubiquitin ligases and can regulate the stability of substrate proteins by increasing or decreasing their ubiquitylation (Lu and Hunter, 2014). It is likely that Pin1 overexpression may lead to the degradation of CAST via the ubiquitin-proteasome pathway in response to glutamate injury. Taken together, this results suggested that Pin1 may be the upstream regulator of the CAST/calpain2 pathway-mediated necrosis through its influence on transcription, translation, and post-translational modification of CAST.

In addition the pro-death roles of Pin1, some other studies reported that Pin1 also play a pro-survival role in neurons, such as, neuronal death associated with the absence of Pin1 in AD (Shen et al., 2009). AD is caused by aggregation of amyloid precursor protein (APP) and microtubule-binding protein tau. Pin1 could bind to p(T/S)-P of p-Tau and pAPP and inhibit the development of p-Tau/p-APP-mediated neurodegeneration by protecting Tau and APP from phosphorylation (Lu et al., 2016). Pin1 has opposite effects in that it could act as a protector or inhibitor for the development of neural diseases. This could be attributed to different disease mechanisms, such as oxidative stress, metabolic dysregulation, inflammation, and ionic dysregulation, which lead to different effects and manifestations (Driver et al., 2015). Similarly, one common biological mechanism might operate inversely in different cell types (Behrens et al., 2009). Thus, further investigations about the genetic and metabolic overlap between different cell types may provide important pathophysiological and therapeutic insights for Pin1-related diseases.

## Conclusion

**FIGURE 8:**
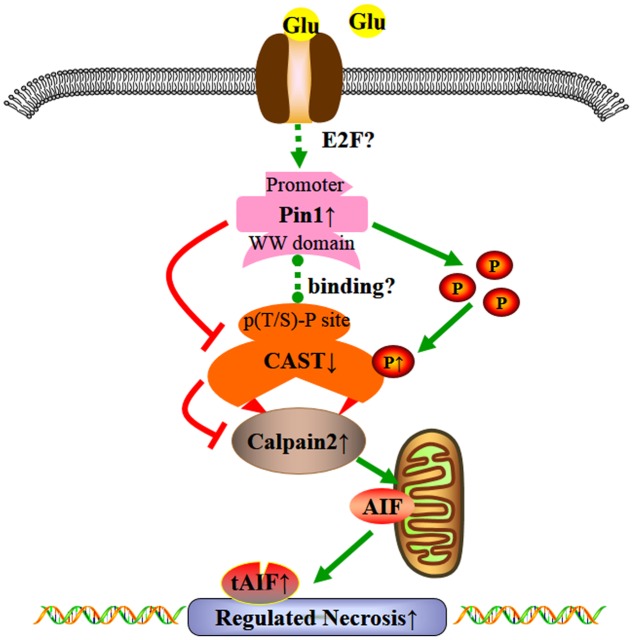
Possible mechanisms underlying the effect of glutamate in regulated necrosis in retinal neurons. The role of Pin1-CAST/calpain2 pathway induced by excessive glutamate in regulated necrosis of retinal neurons.

We demonstrated that the overload of glutamate may lead to regulated necrosis in retinal injury. Excessive glutamate may lead to Pin1-mediated CAST inhibition, resulting in the activation of calpain2 and regulated necrosis in retinal neurons (**Figure 8**). This is a new pathway for neuronal protection in glutamate-induced injury and expands our understanding of the Pin1-CAST/calpain2 neurodegenerative mechanism. Due to the prominent role of glutamate in neurologic diseases (Gu et al., 2014), therapies that inhibit glutamate-induced cell necrosis by Pin1-CAST/calpain2 pathway could be a novel therapeutic targets for the prevention of glutamate excitotoxicity. Finally, since the regulatory mechanism of Pin1-induced by excessive glutamate has not been clearly clarified, this will be the focus of our further research.

## Author Contributions

In this study, KX and JH designed the work. SW conducted the experiments, analyzed the data, and prepared the manuscript and images. LL, MW, YH, YW, ZW, DC, HZ, DJ, and FL helped to conduct the experiments, prepared the manuscript and images, collected and analyzed the data and literature. KX and SW revised the manuscript. All authors read and approved the final manuscript. All authors agreed to be accountable for all aspects of the study in ensuring that questions related to the accuracy or integrity of any part of the work are appropriately investigated and resolved.

## Conflict of Interest Statement

The authors declare that the research was conducted in the absence of any commercial or financial relationships that could be construed as a potential conflict of interest.

## Supplementary Material

**FIGURE S1 |** The complete blot of each antibody used. The blots of Pin1, calpain2, and tAIF showed specific bands at 18, 80, and 57 kDa, respectively. The blots of CAST showed bands at 126, 70, and 25 kDa. The band at 25 kDa were weak, whereas the band at 70 kDa was weak and not visible on all blots. So we quantified the main band at 126 kDa. The blots of PTEN showed mainly bands at 47 kDa.
