# Supplementary figures and images for: Pin1 Promotes Regulated Necrosis Induced by Glutamate in Rat Retinal Neurons via CAST/Calpain2 Pathway
Source: Front Cell Neurosci. 2018 Jan 22;11:425. doi: 10.3389/fncel.2017.00425 (PMC5786546; doi:10.3389/fncel.2017.00425)

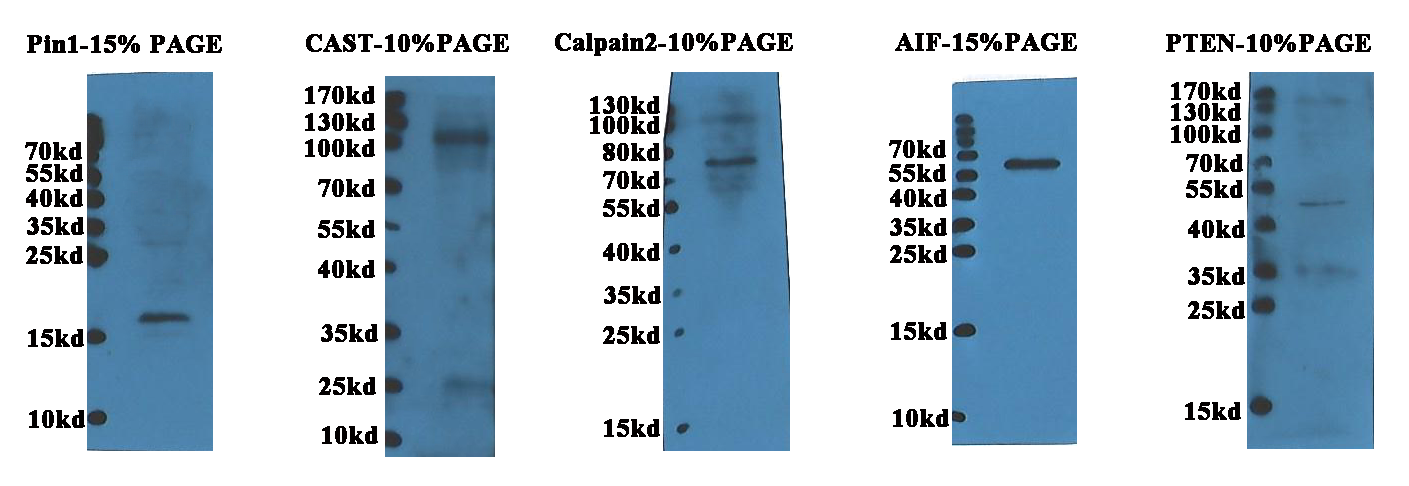

Supplement: Supplementary file 1 [file Image_1.TIF]
